# Premenstrual syndrome incidence rate and risk factors among the working population in the Republic of Korea: a prospective cohort study

**DOI:** 10.1186/s12905-022-01852-5

**Published:** 2022-06-29

**Authors:** Wanhyung Lee, Seunghyun Lee, Joonho Ahn, Ryoon Sun Lee, Seong-Kyu Kang

**Affiliations:** 1 Department of Occupational and Environmental Medicine, Gil Medical Center, Gachon University College of Medicine, 21 Namdong-daero 774 beon-gil, Namdong-gu, 21565 Incheon, Republic of Korea; 2 Department of Occupational and Environmental Medicine, Seoul St. Mary’s Hospital, College of Medicine, The Catholic University of Korea, Seoul, Republic of Korea; 3 Department of Obstetrics and Gynecology, Gil Medical Center, Incheon, Republic of Korea

**Keywords:** Premenstrual Syndrome, Menstrual cycle, Workers, Incidence, Risk factor

## Abstract

**Background:**

Premenstrual syndrome (PMS) is the most common disease of the genitourinary tract in women. Although a sizeable proportion of women have symptoms or diagnosed PMS, its etiology remains unclear. The purpose of this cohort is to offer incidence and relevant risk factors of PMS among reproductive-aged Korean female workers.

**Methods:**

Cohort data used were from the National Health Insurance Service–Female Employees (from 2007 to 2015) conducted by the NHIS. A total of 121,024 female workers were analyzed to estimate the incidence and hazard ratio of PMS. PMS data was based on information obtained from medical facility visits during an eight-year follow-up.

**Results:**

The incidence of PMS was 7.0% during follow-up periods. In industrial classification, human health and social work activities have the highest incidence (9.0%) of PMS. Cumulative incidence of PMS has continuously increased by approximately 1% annually for eight years. Adjusted hazard ratio with 95% confidence interval was significantly higher in the 15–19 years old age group (2.81, 95%CI 2.35–3.36), manual worker (1.06, 95%CI 1.01–1.12), with anemia (1.13, 95%CI 1.06–1.20), and underweight (1.21, 95%CI 1.10–1.25) compared to those in the reference group.

**Conclusion:**

This study describes the PMS status with trend and risk factors using follow-up design among women under a middle-aged working population. Further study is warranted for better understanding on the risk factors of PMS for reproductive-aged female workers.

## Background

Premenstrual syndrome (PMS) or premenstrual tension syndrome is the most common disease of the genitourinary tract in women of reproductive age during the luteal phase of the menstrual cycle (one week before menstruation) [1]. Its symptoms included one or more emotional or physical symptoms characterized by recurrent, moderate-to-severe affective behavioral symptoms, which disappear within few days of menstruation. Although 50%–80% of reproductive-age women have more than one symptom of PMS and approximately 30%–40% of women had been diagnosed with PMS, the etiology of PMS is still unclear [2, 3].

PMS is closely linked to an increased risk of psychiatric disease and decreased quality of life resulting from poor physical health and high psychological distress [4, 5]. Furthermore, previous biochemical studies revealed that PMS is not only a symptom-based disease but is also related to immune system and whole-body inflammation, which could trigger chronic and severe diseases [6, 7].

PMS could increase vulnerability in women, especially the working population. Workers with PMS experienced low work-related quality of life, frequent work absenteeism, and reduced work productivity [8, 9]. It could be negative of workability or working sustainability. Moreover, PMS could also be exacerbated from work-related factors. Work stress and excessive responsibility in the workplace is associated with an increased risk of PMS [10]. Some working women also had no enough time for medical facility visits due to work circumstances (long working hours or shift of work) [11], especially for a diseases that are not life-threating, such as PMS. Working might be closely associated with PMS as bidirectional causal effect.

However, to date, the impact of work-related factors on PMS remains unknown. Thus, we conducted this study for incidence and risk factors of PMS among reproductive-aged Korean female workers using a national representative cohort data.

## Methods

### Data and study participants

**Fig. 1:**
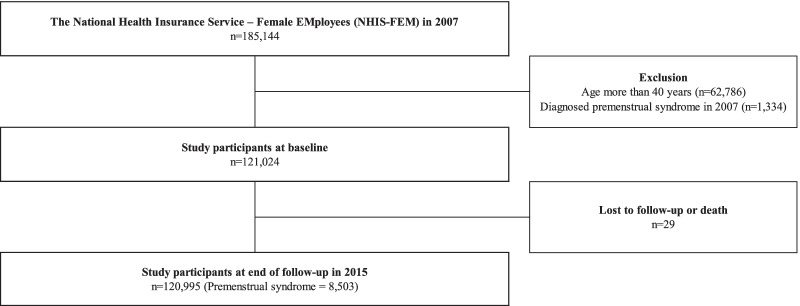
Schematic of study participants

In this study, we utilized data from the National Health Insurance Service–Female Employees (NHIS-FEM) (2007–2015) conducted by the NHIS in South Korea. The Korean National Health Insurance (KNHI) program provides mandatory public health insurance, covering medical care services, which consisted of national health insurance, medical aid, and long-term care insurance, to the entire population residing within the Korean territory [12, 13]. The NHIS established a cohort to provide national representative and valuable information for professionals of public health or policy makers with data from health insurance and health examinations. The NHIS-FEM was based on the NHIS cohort. In general, to analyze the health status and major disease status on the working population of Korean women, the NHIS extracted about 5% (185,144) of 3.71 million people aged 15–64 years (economic activity population) who are qualified in the KNHI by the end of December 2007. It is a non-identifiable data obtained by the cohort form, including qualification and income information (social economic variables), medical and hospital use history, health examination results, and workplace information from 2007 to 2015. We selected young and middle-aged workers aged between 15 and 39 years old from a total 185,144 subjects in 2007 to demonstrate the risk and incidence of PMS. To identify the incidence of PMS, we set a one-year wash-out period for PMS that excluded subjects diagnosed with PMS in 2007. Finally, 108,541 workers were selected for the current study. Detailed information was demonstrated in Fig. 1.

### PMS

PMS defined as medical and hospital facility visit history, which were categorized in accordance with standardized protocol of the Korea Classification of Diseases and Causes of Death, 4^th^ edition, which corresponds to the International Classification of Diseases, 10th revision (ICD-10) [14]. PMS was classified in the “N94 Pain and other conditions associated with female genital organs and menstrual cycle” in the ICD-10. N94 included nine sub-diseases codes, as follow: N94.0, mittelschmerz; N94.1, dyspareunia (excluding psychogenic dyspareunia (F52.6)); N94.2, vaginismus (excluding psychogenic vaginismus (F52.5)); N94.3, premenstrual tension syndrome; N94.4, primary dysmenorrhea; N94.5, secondary dysmenorrhea; N94.6, dysmenorrhea (unspecified); N94.8, other specified conditions associated with female genital organs and menstrual cycle; and N94.9, unspecified condition associated with female genital organs and menstrual cycle.

### Socioeconomic and occupational characteristics

Socioeconomic status was based on baseline characteristics of the initial cohort year (2007). It was classified according to three age groups (15–19, 20–29, and 30–39 years old) and four household income groups (low, middle–low, middle–high, and high). Occupational characteristics included International Standard Industrial Classification (ISIC), type of work, and duration of work (< 6, 7–12, and > 12 month). According to a previous report [15], the type of work was categorized based on the International Standard Classification of Occupations (ISCO) for organization of occupations. Office workers were legislators, managers, professionals, senior officials, associated professionals, and technicians and manual workers were sales workers, clerks, customer service workers, assemblers, craft workers, plant and machine operators, and elementary workers. Other workers were agriculture, fishery, and forestry.

### Health and behavioral characteristics

Health and behavioral characteristics were based on the first result from the national health screening gathered during follow-up periods. In Korea, all workers were requested to undergo national health screening at least annually or biannually according to occupation. The questionnaires during national health screening obtained information on one’s medical history, medication, and behavioral health. We used questionnaires for hypertension, diabetes, anemia, body mass index (BMI), and smoking and drinking habituation. BMI was categorized into three groups based on Asian standard, as follows: underweight (< 18.5 kg/m^2^), normal weight (< 25 kg/m^2^), and obese (≥ 25 kg/m^2^). Smoking status was classified into three groups (never, past, and current). Current smokers defined as those who had smoked more than 100 cigarettes (five packs of cigarettes) during their lifetime and were currently smoking. Never smokers were those who had smoked less than 100 cigarettes (five packs of cigarettes) in their lifetime. Past smokers were individuals who had smoked in the past but are no currently smoking. Severe drinking included those who had alcohol drinking of more than five glasses, twice per week.

### Statistical analysis

We conducted analysis for the incidence of PMS according to baseline characteristics, health status, and behavioral health using the chi-squared test. Incidence and cumulative incidence of PMS were calculated according to the ISIC and follow-up year. The hazard ratio (HR) and 95% confidence intervals (CI) were estimated using the Cox regression analyses of PMS with adjusted covariates. For the proportional hazard assumption, a regression model of scaled Schoenfeld residuals against time was assessed for zero slope and a p value was found to be greater than 0.1, indicating the proportional hazards assumptions for PMS was satisfactory. All analyses were conducted using SAS version 9.4 (SAS Institute, Cary, NC, USA).

## Results

A total of 121,024 female workers participated in this study, of which 8,503 (7.0%) workers have PMS. The baseline characteristics of study participants according to PMS are illustrated in Table 1. Of the 8,503 workers suffering from PMS, the categories with the highest proportion were as follows: women aged 20–29 years old (67.7%), with low household income (39.3%), office type of workers (53,3%), and those working > 12 h (62.5%).

**Table 1:** Baseline characteristics of young and middle-aged Korean workers in 2007 according to premenstrual syndrome during follow-up period

	Total participants, *n* (%)	Premenstrual syndrome, *n* (%)		*P*-value
		No	Yes	
Total participants	121,024 (100.0)	112,521 (100.0)	8,503 (100.0)	
*Socioeconomic status*				
Age				< 0.0001
15–19	1,459 (1.2)	1,279 (1.1)	180 (2.1)	
20–29	64,541 (53.3)	58,788 (52.2)	5,753 (67.7)	
30–39	55,024 (45.5)	52,454 (46.6)	2,570 (30.2)	
Household income level				< 0.0001
Low	47,865 (39.5)	44,522 (39.6)	3,343 (39.3)	
Middle–low	32,578 (26.9)	30,119 (28.9)	2,459 (28.9)	
Middle–high	24,661 (20.4)	22,840 (21.4)	1,821 (21.4)	
High	15,920 (13.2)	15,040 (10.3)	880 (10.3)	
*Occupational characteristics*				
Type of work				< 0.0001
Office	65,236 (53.9)	60,701 (53.9)	4,535 (53.3)	
Manual	37,003 (30.6)	34,219 (30.4)	2,784 (32.7)	
Other	18,785 (15.5)	17,601 (15.6)	1,184 (13.9)	
Duration of work (month)				0.0003
< 6	15,033 (12.4)	13,931 (12.4)	1,102 (13.0)	
7–12	27,739 (22.9)	25,655 (22.8)	2,084 (24.5)	
> 12	78,252 (64.7)	72,935 (64.8)	5,317 (62.5)	

Table 2 demonstrates the health and behavioral characteristics of female workers who responded to the national health screening with or without PMS. It was determined that 7,486 (7.2%) respondents among workers who have PMS underwent national health screening. In categories of health status, a higher percentage of female workers who suffer from PMS than non-PMS workers were as follows: workers with anemia (18.3%) and underweight (17.6%). According to behavioral health, current smokers (4.6%) account for a higher proportion of PMS workers than non-PMS workers.

**Table 2:** Health and behavioral characteristics of young and middle-aged Korean workers who participated in the national health screening according to premenstrual syndrome during follow-up period

	Total participants, *n* (%)	Premenstrual syndrome, *n* (%)		*P*-value
		No	Yes	
National health screening participants	104,574 (100.0)	97,088 (100.0)	7,486 (100.0)	
*Health status*				
Hypertension				< 0.0001
No	101,828 (97.4)	94,485 (97.3)	7,343 (98.1)	
Yes	2,746 (2.6)	2,603 (2.7)	143 (1.9)	
Diabetes				0.0064
No	103,615 (99.1)	96,176 (99.1)	7,439 (99.4)	
Yes	959 (0.9)	912 (0.9)	47 (0.6)	
Anemia				0.0445
No	86,150 (82.4)	80,036 (82.4)	6,114 (81.7)	
Yes	18,424 (17.6)	17,052 (17.6)	1,372 (18.3)	
Body Mass Index				< 0.0001
Underweight (< 18.5)	14,483 (13.9)	13,166 (13.6)	1,317 (17.6)	
Normal weight (18.5–24.9)	77,848 (74.4)	72,328 (74.5)	5,520 (73.7)	
Obesity (≥ 25)	12,243 (11.7)	11,594 (11.9)	649 (8.7)	
*Health behavioral*				
Smoking				0.0094
Never or past	100,199 (95.8)	93,057 (95.8)	7,142 (95.4)	
Current	4,375 (4.2)	4,031 (4.2)	344 (4.6)	
Drinking				0.9255
Non-severe	100,922 (96.5)	93,696 (96.5)	7,226 (96.5)	
Severe	3,652 (3.5)	3,392 (3.5)	260 (3.5)	

**Fig. 2:**
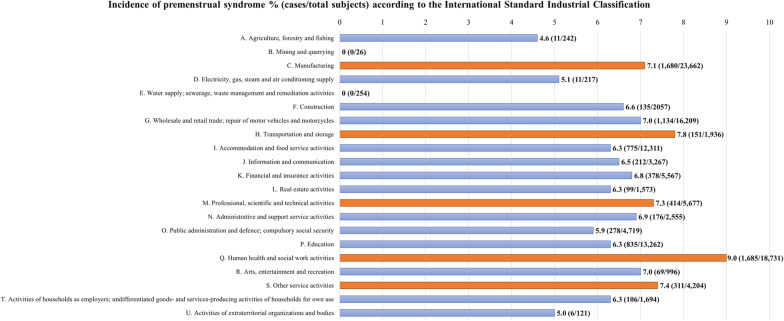
Incidence of premenstrual syndrome (%) according to International Standard Industrial Classification

As illustrated in Fig. 2, according to the ISIC, workers in human health and social work activities had the highest incidence of PMS (9.0%). Subsequently, incidence of PMS was over 7.0% in workers on transportation and storage (7.8%); professional, scientific, and technical jobs (7.3%), and manufacturing (7.1%).

**Fig. 3:**
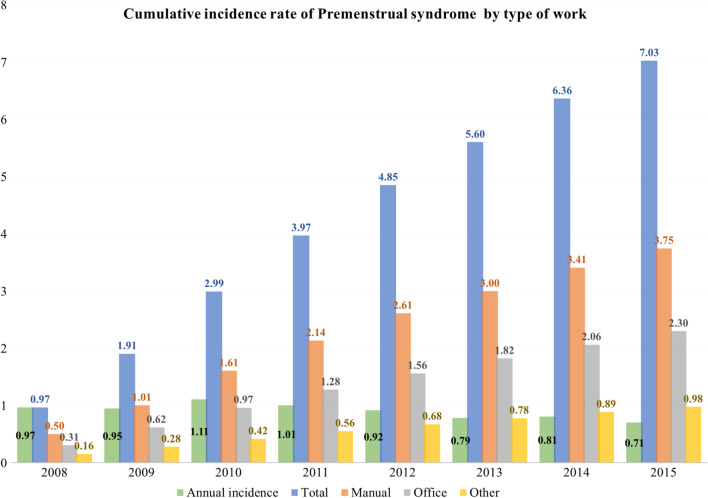
Cumulative incidence of premenstrual syndrome during follow-up periods

Figure 3 showed an eight-year cumulative incidence of PMS by working type. Cumulative incidence has continuously increased by approximately 1% annually. Manual workers presented highest incidence rate during follow-up periods compared with office or other workers.

The multivariate Cox regression result of PMS is presented in Table 3. The Cox regression model was adjusted for age, household income level, working type, duration of work, health status (hypertension, diabetes, anemia, and BMI), and behavioral health (smoking and drinking status). After adjustment, the HRs of aged 15–19 years old (HR [95% CI], 2.81 (2.35, 3.36)), manual workers (HR [95% CI], 1.06 (1.01, 1.12)), and workers with work duration of 7–12 h (HR [95% CI], 1.06 (1.00, 1.13)) significantly increased compared to those in the reference group. HRs of female workers who have anemia and were underweight were higher than the reference group (HR [95% CI], 1.13 (1.06, 1.20), 1.21 (1.10, 1.25), respectively). Moreover, HR associated with smoking was 1.21 (1.02, 1.39) of workers who had smoked in the past.

**Table 3:** Results from the multivariate Cox regression analyses of premenstrual syndrome among national health screening examined participants (n = 104,574)

	Premenstrual syndrome, Hazard ratio (94% confidence interval)	
	Crude	Multivariable
*Socioeconomic status*		
Age		
15–19	**2.87 (2.45–3.37)**	**2.81 (2.35–3.36)**
20–29	**2.00 (1.90–2.10)**	**1.95 (1.85–2.06)**
30–39		Reference
Household income level		
Low	**1.28 (1.19–1.39)**	1.01 (0.92–1.09)
Moderate–low	**1.40 (1.29–1.51)**	1.02 (0.93–1.11)
Moderate–high	**1.36 (1.25–1.48)**	1.08 (0.99–1.18)
High	Reference	Reference
*Occupational characteristics*		
Type of work		
Office	Reference	Reference
Manual	**1.09 (1.04–1.14)**	**1.06 (1.01–1.12)**
Other	**0.90 (0.84–0.96)**	**0.89 (0.82–0.97)**
Duration of work (month)		
< 6	**1.09 (1.01–1.16)**	1.06 (0.98–1.15)
7–12	**1.11 (1.06–1.17)**	1.06 (1.00–1.13)
> 12	Reference	Reference
*Health status*		
Hypertension		
No	Reference	Reference
Yes	**0.71 (0.60–0.84)**	0.89 (0.75–1.05)
Diabetes		
No	Reference	Reference
Yes	**0.67 (0.50–0.91)**	0.86 (0.64–1.15)
Anemia		
No	Reference	Reference
Yes	**1.15 (1.09–1.22)**	**1.13 (1.06–1.20)**
Body Mass Index		
Underweight (< 18.5)	**1.31 (1.23–1.40)**	**1.21 (1.10–1.25)**
Normal weight (18.5–24.9)	Reference	Reference
Overweight (≥ 25)	**0.73 (0.67–0.80)**	**0.82 (0.75–0.89)**
*Health behavioral*		
Smoking		
Never or past	Reference	Reference
Current	**1.11 (1.01–1.25)**	1.03 (0.90–1.15)
Drinking		
Non-severe	Reference	Reference
Severe	0.99 (0.87–1.13)	1.02 (0.82–1.54)

Bold indicated statistical significance

## Discussion

The aim of this investigation was to assess the PMS status and risk factors for PMS among the young-aged working population of Korea. The incidence based on medical facility visit information of PMS was 7.0% based on our investigation during an eight-year follow-up, which is higher than the general population in Korea from a previous study (2.1%) [16]. There was a significantly increased risk of PMS in groups of younger ages, shorter career, manual workers, with anemia, and are underweight.

Initially, as women enter the workplace and deal with social stressors, they increasingly face emotional tension and mental and physical stress. PMS was found to be more prevalent in younger-aged women [17], which is similar to the results from current analysis. Indeed, a prospective cohort study reported that younger-age female workers accounted for about 48% of the high-stress employee group [18]. The high job demands and low job control, such as strong physical and psychological job demands, or low skill discretion and decision authority, can affect occupational stress in younger workers due to characteristics of the labor market.

Work-related stress, occupational risk, and unhealthy work environment have been considered as risk factors for PMS [4, 10, 19]. Our study demonstrated the highest incidence of PMS in “Q. Human health and social work activities” from the ISIC. These results are consistent with those of previous studies. Previous study referring to healthcare workers, such as nurses, have a greater tendency toward genitourinary diseases, including PMS, due to heavy working conditions and stressful work [9, 20, 21].

Manual or blue-collar workers included sales workers, clerks, customer service workers, assemblers, craft workers, plant and machine operators, and elementary workers. These workers could be exposed to various occupational hazard factors (chemicals, noise, ergonomic risk, or personal interaction), unhealthy working circumstance (long working hours, complicated work schedule, or long commuting time), and occupational mental factors (tight working standards, challenge level of work, or tight working standards) more than others [15, 21]. Thus, it can be suggested that working conditions could be key PMS-related factors.

The current analysis indicated a higher risk in underweight group than in the obesity group. This finding is contrary to previous studies which have suggested that obesity might be related to the development of PMS [22, 23]. However, previous analyses were conducted on the association between obesity or elevation of weight and PMS based on general population. Thus, findings of this study suggest that underweight could also be an important risk factor for PMS, at least among working population. Previous research demonstrated weight misperceptions and higher likelihood of weight control attempts in female workers, even for under or normal weight, due to social and working activity [24, 25].

There is very little known on the association between anemia and PMS. Based on the result of this study that anemia as a risk factor of PMS suggests that PMS is closely related to hematological effect. Previous study revealed both higher risk and severity of PMS in anemic group than in non-anemic group [26]. The etiology of PMS remains unclear and could be a multifactorial event. Some evidence suggests that PMS is related to serotonin imbalance [27]. Anemia due to iron deficiency is related to the decreased neurotransmitter function, such as serotonin [28]. Decreased neurotransmitter level might be linked to iron deficiency anemia, which is related to symptoms of PMS. The association between hematologic physiology and developing PMS could be an important issue for future research.

The current analysis demonstrated risk factors of PMS such as younger, manual workers, anemia, and underweight women. These factors could increase the risk of PMS with interactive effect each other. For example, it is well-known that underweight correlated with anemia, these factors could make worse PMS together. Further researches require to warrant the interaction effect among risk factors from current study on PMS.

Although this study has shown that PMS status with trend and statistically significant risk factors, using national representative data with the large sample size of working population and follow-up design focused on reproductive-age group, this study was limited to the absence of clinical diagnosis of PMS. All data from the Korean NHIS database were based on information from medical facility visits and were recorded in ICD-10 code format. We present PMS based on severe cases due to the nature of data. Thus, these findings may not be sufficient enough to be generalized to the whole spectrum of PMS. However, patient records, involving severe medical disease spectrum, were considered reliable in a previous study that used data from the NHIS dataset [29]. Our study was also limited by the lack of information on history, working conditions; medical and lifestyle factors, such as shift work, working hours, workplace circumstance, hysterectomy history, contraceptives use, other medication history, exercise level; and dietary factors which might have influenced the development of PMS.

## Conclusion

In conclusion, the main goal of the current study was to describe PMS status and risk factors among middle-aged working population of Korea. The incidence of PMS among workers is higher than the general population. The risk of developing PMS is increased by occupational characteristics (ISIC, ISCO, and career duration), health status (anemia and underweight), and lifestyle factors (smoking).

## Data Availability

The datasets analysed during the current study are available from the NHIS data sharing service data (https://nhiss.nhis.or.kr/bd/ab/bdaba000eng.do).
